# Effects of Minocycline Hydrochloride as an Adjuvant Therapy for a Guided Bone Augmentation Procedure in The Rat Calvarium

**DOI:** 10.3390/dj11040092

**Published:** 2023-03-31

**Authors:** Bob Biewer, Eric Rompen, Michel Mittelbronn, Gaël P. Hammer, Pascale Quatresooz, Felix Kleine Borgmann

**Affiliations:** 1 Department of Periodontology and Oral Surgery, Faculty of Medicine, 4000 Liège, Belgium; 2 National Center of Pathology (NCP), Laboratoire National de Santé (LNS), 3555 Dudelange, Luxembourg; 3 Department of Oncology (DONC), Luxembourg Institute of Health (LIH), 1445 Strassen, Luxembourg; 4 Luxembourg Centre for Systems Biomedicine (LCSB), University of Luxembourg, 4367 Esch-sur-Alzette, Luxembourg; 5 Luxembourg Center of Neuropathology (LCNP), 3555 Dudelange, Luxembourg; 6 Faculty of Science, Technology and Medicine (FSTM), University of Luxembourg, 4367 Esch-sur-Alzette, Luxembourg; 7 Department of Life Science and Medicine (DLSM), University of Luxembourg, 4365 Esch-sur-Alzette, Luxembourg; 8 Department of Human Histology and Dermatopathology, University Hospital of Liège University (CHU), 4000 Liège, Belgium

**Keywords:** cortical perforation, guided bone regeneration, minocycline, vertical bone augmentation

## Abstract

This in vivo study reports the influence of minocycline-HCl administration on extra-skeletal bone generation in a Guided Bone Augmentation model, utilizing titanium caps placed on the intact as well as perforated calvaria of rats. The test group was administered 0.5 mg/mL minocycline-HCl with the drinking water, and the amount of bone tissue in the caps was quantified at three time points (4, 8 and 16 weeks). A continuously increased tissue fill was observed in all groups over time. The administration of minocycline-HCl as well as perforation of the calvaria increased this effect, especially with regard to mineralization. The strongest tissue augmentation, with 1.8 times that of the untreated control group, and, at the same time, the most mineralized tissue (2.3× over untreated control), was produced in the combination of both treatments, indicating that systemic administration of minocycline-HCl has an accelerating and enhancing effect on vertical bone augmentation.

## 1. Introduction

The management and treatment of bone defects is a major clinical problem in periodontology and oral implantology. While various established and predictable methods exist for alveolar ridge augmentation, these procedures remain challenging, with a significant incidence of complications and failures [1,2]. The guided bone regeneration procedure is one of the most promising surgical techniques to increase limited alveolar bone for implant placement. This principle can be applied to generate new bone formations on the adjoining bone surface, beyond the skeletal boundaries [3,4]. Different kinds of barrier membranes and resorbable or non-resorbable materials, such as titanium, are used to achieve this guided bone augmentation (GBA) [5]. For severe three-dimensional reconstructions, however, non-resorbable membranes are the most effective [2,6]. The close spatial and temporal correlation between angiogenesis and bone formation, as well as the importance of blood supply in guided bone generation, have been demonstrated [3,7,8]. Some authors have emphasised the opening of the marrow spaces to obtain a bleeding surface [3,9,10], whereas others have demonstrated that bone regeneration from a non-injured cortical layer may also be possible [11,12].

Postoperative complications risk jeopardising the outcome. These can include tissue necrosis with wound exposure and especially bacterial infections, arising from not only the external environment but also adjacent structures such as the sinuses, nasopharynges and oral cavity [13]. The inflammatory process activates the host defence system in an overshooting manner, with increased production of cytokines progressing towards alveolar bone resorption and irreversible bone loss [14].

Tetracyclines have a broad antibacterial spectrum, being effective against gram-positive and gram-negative bacteria, spirochetes and rickettsia [15]. These antibiotics have long been used as adjuncts in treating periodontal diseases [16,17]. Minocycline hydrochloride is a semi-synthetic tetracycline derivative showing particularly high concentrations in the gingival crevicular fluid [18,19,20]. Its anti-inflammatory properties [19,21,22], modulatory effects on the biology of periodontal fibroblasts [23,24] and efficiency against plaque microorganisms [25] make minocycline useful in managing periodontal diseases. In addition to its proven efficiency in periodontal therapy, minocycline has displayed a high affinity towards mineralised tissues [15], with positive effects on bone regeneration [20]. Tetracyclines have been regarded as one of the only antibacterial agent classes with positive effects on bone tissue remodelling and healing [26]. Multiple studies have demonstrated that by non-antimicrobial, anti-collagenolytic mechanisms, tetracyclines can improve bone mass by inhibiting osteoclast-mediated bone resorption and enhance bone formation through increased osteoblastic activation and upregulation of protein synthesis [27]. They stimulate osteogenesis and apoptosis of osteoclasts and inhibit inflammatory bone resorption and osteoclast genesis [28,29].

The direct action of tetracyclines on osteoclasts is a limitation of their resorption activities by reducing the proton pump and the sealing zone delineated by the ruffed border [23], as well as inhibition of cysteine proteinases and/or matrix metalloproteinases, resulting in the inhibition of osteoclastic bone resorption [30].

Tetracyclines also indirectly influence bone resorption processes by inhibiting the osteoblast collagenase synthesis necessary for non-calcified osteoid degradation [31], preventing direct access of osteoclasts to the underlying mineralised bone surface and its resorption. Systemic tetracyclines also suppress osteoclast recruitment following surgery [32]. Moreover, it has been shown in vivo that tetracyclines could stimulate bone formation [33] and directly influence mineralisation processes by increasing osteoblast alkaline phosphatase synthesis [34,35]. Golub et al. reported that oral administration in a standard animal model of postmenopausal osteoporosis, the ovariectomised aged rat, dramatically reduced the severity of skeletal (tibial) bone density loss and reduced alveolar (periodontal) bone loss, demonstrating that tetracyclines can inhibit both oral and systemic bone loss [36].

Despite the verified pro-anabolic and anti-catabolic properties of minocycline on bone tissue, to the best of the authors’ knowledge, therapy has never been assayed within a GBA approach using empty and occlusive regeneration chambers.

In this histomorphological work in the rat calvaria, we aimed to analyse the influence of oral administration of minocycline in combination with cortical perforations on the amount and structure of the expected new bone formations. We chose to investigate this molecule to improve the outcome and predictability of GBA procedures.

## 2. Material and Methods

### 2.1. Animal Preparation and Surgical Procedures

This study used 20 adult male Wistar rats (8 weeks old with a bodyweight of 340 g ± 20 g). All animal experimental procedures were conducted under ethical clearance, which was reviewed by the Institutional Animal Care and Use Ethics Committee of the University of Liège, Belgium (26112019). Animal Research Reporting of In Vivo Experiments (ARRIVE) guidelines were carefully followed, as was national and European legislation [37]. The rats were obtained from the animal centre of Liège University and kept in separate cages in standard laboratory room conditions (average 24.8 °C, 55–70% humidity) on a 12 h light–dark cycle in the University Animal Facility. The animals were given free access to water and a standard laboratory diet.

For the surgical procedure, rats were anesthetised by intramuscular injection of a combination of atropine sulphate (Stella, Chênée, Liège, Belgium; 0.25 mg/kg), xylazine 2% (Rompun^®^, Bayer AG, Barmen, Germany; 0.5 mL/kg) and ketamine (Imalgène^®^ 500, Rhone Mérieux, Lyon, France; 50 mg/mL). The frontal–parietal region was shaved and disinfected with denaturalised alcohol (70% ethylic alcohol, 30% ether). All surgery was performed under aseptic conditions. A midsagittal incision was made through the skin tissues and periosteum. A full-thickness flap was raised to completely expose the parietal bone; the surgical area was kept moist by a saline-filled sterile gauze. Two titanium caps (self-made from sheer titanium metal sheets, finished dimensions 6 × 4 × 3 mm) were placed, one on each side of the sagittal median skull suture, and strongly anchored laterally to the periosteum (Seraflex^®^ 4.0; Serag-Wiessner, Naila, Germany) before being covered with the periosteal cutaneous flap.

**Figure 1:**
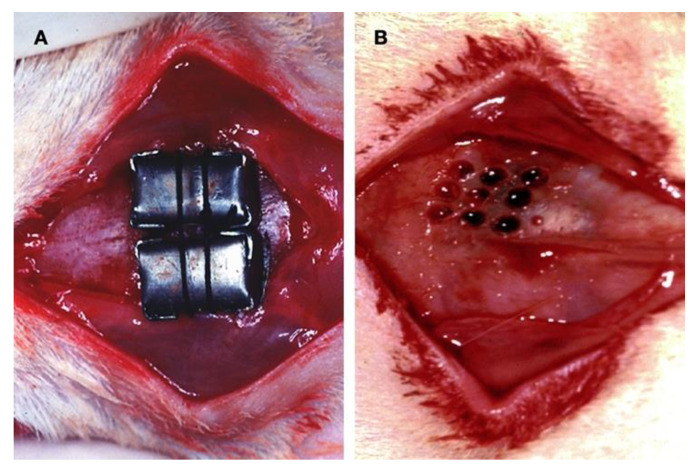
Surgical procedure: (**A**) Custom-made, smooth-surface, stiff caps of pure titanium were used as a barrier. The space inside was left empty for the graft to achieve bone augmentation. (**B**) The decortication procedure consisted of drilling nine holes into the outer layer of the cortical bone to induce active bleeding from the marrow space at the experimental sites.

On the left side of the calvaria, the cap was placed on the intact periosteum-free cranial bone. On the right side, the cortical bone plate of the calvaria was perforated before placement. Nine cortical perforations were made with a round tungsten carbide bur #8 under copious saline irrigation to create a surgically induced standardised calvaria bone wound (Figure 1). Care was taken not to damage the dura or brain. Animals were monitored continuously until they had completely recovered from anaesthesia. For the first ten days after surgery and at least until wound healing was complete, animals were monitored at least once daily by the animal care takers and experimentalists using a scoring-checklist indicating weight, food and water consumption, wound healing, appearance and activity.

The surgical protocol being identical, the animals were randomly divided into two groups: one without further treatment and the other with minocycline administered systemically by adding 0.5 mg/mL to the drinking water. Three animals of each group (*n* = 3 animals in 2 groups with two inserts each, resulting in a total of 12 surgical sites per time-point) were euthanised 4- and 8-weeks post-surgery by injection of pentobarbital after sedation with ketamine and xylazine. The remaining rats (*n* = 4) were sacrificed after 16 weeks, and tissue was collected for analysis.

Four series were available for comparative histomorphometrical and histological analyses:

- Control group (C): no calvaria manipulations; no drugs.
- Test group I (P): cortical perforations; no drugs.
- Test group II (MIN): no bone wounding; minocycline-fed.
- Test group III (MIN + P): cortical perforations; minocycline-fed.

### 2.2. Histological Preparation

The specimens were extracted, and the calvaria blocks were excised and fixed in a Bouin solution (3 parts saturated picric acid and 1 part formaldehyde 35%) for 10 days. After decalcification in an EDTA solution at 0.2 mM (pH 7.4), the samples were dehydrated in ascending concentrations of ethanol series for 96 h and embedded with paraffin. The paraffin blocks were archived for experimental use and retrieved for analysis at a later time point (surgery 1995/96, retrieval 2018).

At four locations, 0.5 mm distant from each other, three vertical sections were cut using a tungsten carbide microtome (Supercut 2065, Jung, Germany). One of each was stained with solochrome cyanine, toluidine blue and alkaline phosphatase, respectively, for observation of metal deposits, histomorphometric measurements and histological analyses [38].

### 2.3. Microscopic Analysis

Images of the sections were captured with a 5-megapixel microscope camera (Axiocam 305 colour, Zeiss AG, Oberkochen, Germany) connected to a binocular optical laboratory microscope (Axio Lab.A1, Zeiss AG, Germany). The microscopic analysis was performed quantitatively and qualitatively.

Computer-assisted detailed measurements of the newly generated tissue (ImageJ 2.0, America National Institutes of Health, Bethesda, MD, USA) were made on four solochrome-stained serial sections at ×2.5 magnification to measure the cranial vault’s thickness under the test caps. The results of this histomorphometric quantification were expressed as percentage increases of the cranial vault, with 100% corresponding to a doubling.

Measurements for typing the different tissues and their relative quantification within the newly generated tissue were made on four randomly chosen regions of interest at ×20 magnification. The following tissue components were measured for the total area of the region of interest:

- Newly formed lamellar bone and osteoid tissue (%);
- Woven bone (%);
- Bone marrow (%);
- Connective tissue (%);
- Granulation tissue and blood clot (%);
- Number of osteocytes (Oc) per mm^2^:

The osteocyte density was scored for the mature native skull bone as well as for the newly formed osseous tissue. Considering that osteocytes are the terminally differentiated cells of the osteoblast lineage, this cell counting included all phases of development in osteocyte biology through the different stages of ossification and mineralisation present at the three observation times.

- Number of osteoblasts (Ob) per mm:

The evaluation was limited to those osteoblasts that were found to be lined up in cell fronts (if present in the sample). Scoring the osteoblast density allowed identifying any changes in their size, shape and arrangement over time.

- Number of blood vessels in the newly formed tissue (capillaries per mm^2^);

Detailed histological observations were done at ×40 microscopic magnification.

### 2.4. Statistical Analysis

All quantitative data are expressed as mean ± standard deviation (SD). The four groups were compared by an analysis of variance (multivariable linear models, including the weeks after the intervention, bone perforation, minocycline and their two-way interaction as explanatory variables) to show the variance among and between groups. All statistical analyses were performed by R statistical analysis software (R Project version 4.0.2, Vienna, Austria) [39], and the level of significance was set to 5%.

## 3. Results

Anaesthesia was well-survived by all animals, and healing of the surgical areas was uncomplicated, with no evidence on dissection of adverse reactions or infection. The devices were clinically stable with intact sutures covered by strong fibrous connective tissue and intimately integrated at their edges into the host skull bone. The removal of the test chambers was difficult, especially for the later specimens, because of the new bone formation along their outside. All caps could be manually removed after exposure by dissection without evidence of damage to the tissue contained. New tissue formation was observed in all control and test sites after removal of the covering capsules.

### 3.1. Four-Week Specimens

**Figure 2:**
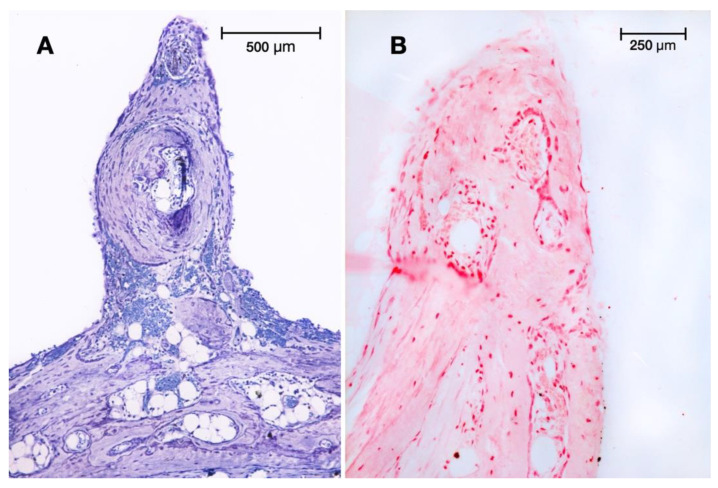
Peaks at 4: (**A**) Spine pointing out of the newly generated tissue in test group I at 4 weeks (cortical perforations). Toluidine blue staining (original magnification: 10×). (**B**) Similar peak observed in test group II (minocycline-treated) at 4 weeks. Alkaline phosphatase (ALP) staining was mainly localised to osteoblasts (original magnification: 20×).

In most control and test sections, the tissue growth had mainly developed close to the walls of the test devices, while the central parts of the chamber were often found empty. A loose and unmineralised connective tissue layer rich in blood vessels covered the generated tissue. Thin peaks mainly composed of a vascular axis with accompanying undifferentiated connective tissue cells were observed pointing out with a height of around 1 to 2 mm from the otherwise flat surface (Figure 2). These peaks were present in both groups at this time point. A blood vessel count was used to determine the capillary density to evaluate the extent of angiogenesis in the newly formed tissue (Table 1). The number of new capillaries, varying between 124 and 140 per mm^2^, was slightly higher in the perforated groups. The number of scored osteoblasts was constant (46–48 Ob per mm), with a size around 20–22 µm in all groups. The osteocyte density (number of osteocytes embedded per mm^2^) in the newly formed lamellar bone was approximately the same in all groups (mean values ranging from 1106 to 1203 per mm^2^, i.e., three times more osteocytes than in the native skull bone, 334–342 Oc per mm^2^).

The control sites presented an increase of the cranial vault with 65.59 ± 15.53% of newly formed tissue, compared to a 99.29% ± 7.60% increase in test group II (minocycline addition), where both groups had no surgical trauma except the cylinder placement (Table 2). Test group I (cortical perforations) presented 73.48% ± 14.99% new tissue formation, which was not statistically different from the control (C). Significant tissue growth of 111.63% ± 9.55% (*p* < 0.001) was found in group III, where both techniques were combined (cortical perforations with minocycline addition). The generated tissue in all samples was mainly composed of three types of tissues: lamellar bone, woven bone and connective tissues, as well as small blood clot and granulation tissue remnants. Cellular debris, artefacts, hyaline structures and empty spaces were not scored and were excluded from calculations.

**Figure 3:**
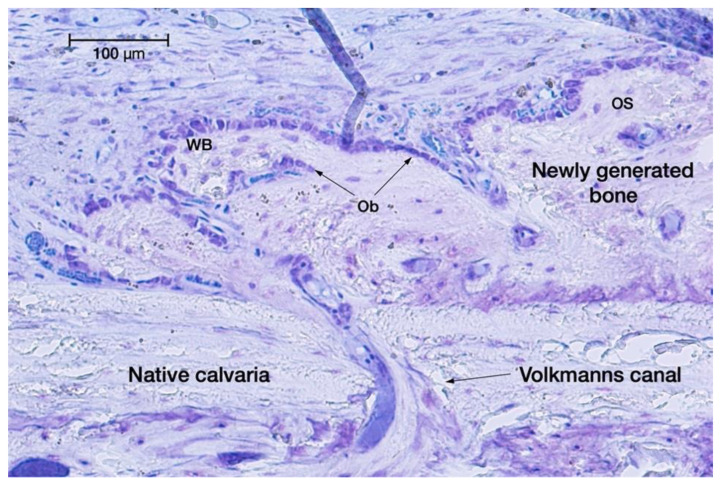
Spontaneous opening of a Volkmann’s canal, letting osteogenic cells migrate from the medullary spaces towards the native skull bone into the regeneration chamber for extra-skeletal new bone formation. Toluidine blue staining (original magnification: 20×).

Lamellar bone developed first at the basal area of the regeneration chambers, close to and contacting the original calvaria bone plate. No enhanced bone-forming activity was observable facing the surgical perforations of the external cortical bone plate in test groups I or III. In some of the non-perforated specimens of group C and test group II, we observed some spontaneous openings of the original cortical bone plate, probably due to widening of some Volkmann’s channels damaged when lifting the periosteum (Figure 3).

**Figure 4:**
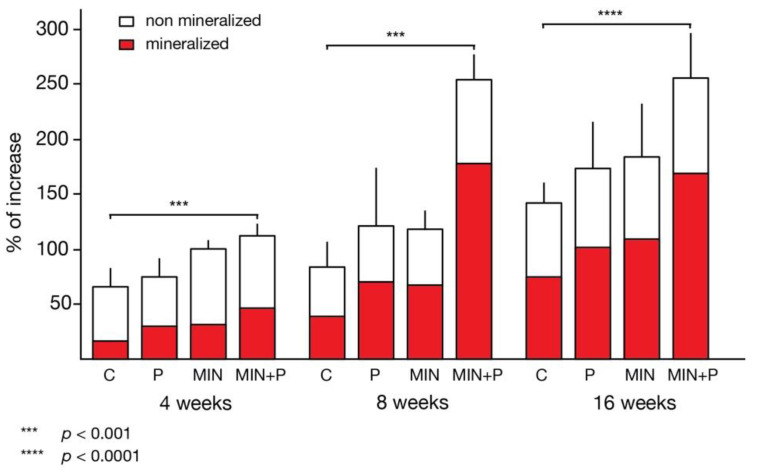
Increase in bone thickness over time. Both the perforation procedure and minocycline administration resulted in an increase in bone thickness, with the strongest effect in the combination of both. This effect was observable at all time points. A large percentage of the newly generated bone was mineralised (red colour; *** *p* < 0.001; **** *p* < 0.0001).

The percentage of lamellar bone was lower in the two non-perforated groups (C: 12.99 ± 2.81%; test group II: 17.36 ± 5.99%) than in the perforated groups (test group I: 22.16 ± 7.47%; test group III: 31.84 ± 9.55%). A similar distribution was found for woven bone: non-perforated groups C 10.24 ± 2.58% and II 12.79 ± 4.20% vs. perforated test groups I 17.31 ± 3.91% and III 16.27 ± 5.20%. The higher values of woven bone indicate more advanced maturity of bone formation. Early bone marrow structures were visible in some specimens. At this point, these were more pronounced in the groups treated with minocycline (test group II: 1.19 ± 1.72%; test group III: 3.41 ± 3.29%) and up to 400% higher than in the two non-treated groups (C: 0.36 ± 0.65%; test group I: 0.69 ± 1.15%), but remained marginal on the whole. The higher bone marrow values in the minocycline groups also indicate more advanced organisation of the already formed bone. The detailed histomorphometric quantifications of the different structures and tissues are summarised in Table 2. Overall, the perforated groups showed a larger extent of mineralisation (test group I: 40.16 ± 9.86%; test group III: 51.51 ± 9.06%) compared to the unperforated control group (23.59 ± 4.04%) and test group II (31.33 ± 6.44%; Figure 4).

Connective tissue, including zones of woven bone, mainly grew in the most superficial regions. These secondary centres of newly mineralised woven bone were covered by osteoid formations and lining osteoblasts, indicating expanding and remodelling activities of the primary woven bone scaffold into lamellar bone, with the proportions of woven bone in the perforated groups significantly greater than in the non-perforated groups. A few osteoclasts were encountered. New bone appositions had also formed to a varying extent at the outer surface of the chambers, especially adjacent to their edges, where the periosteum had been lifted to cover the devices.

### 3.2. Eight-Week Specimens

From 4 to 8 weeks, increases in the generated tissue were seen in all samples, with 82.91 ± 21.86% newly generated tissue under the control devices. The tissue filling in the chambers of test groups I and II was similar: the regenerated tissue area increased 120.65 ± 52.12% and 116.94 ± 17.39%, respectively. A statistically highly significant amount of new tissue formation occurred in test group III (253.6 ± 21.66%; cortical perforations and minocycline-treated), representing more than double the augmentation rate in the other groups.

**Figure 5:**
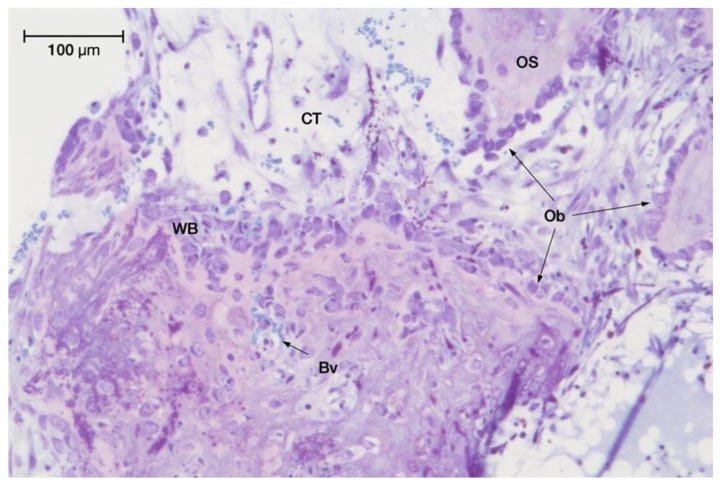
Intense ossification activity observed in minocycline-treated series (test group II) at 8 weeks with toluidine blue staining (original magnification: 20×).

**Figure 6:**
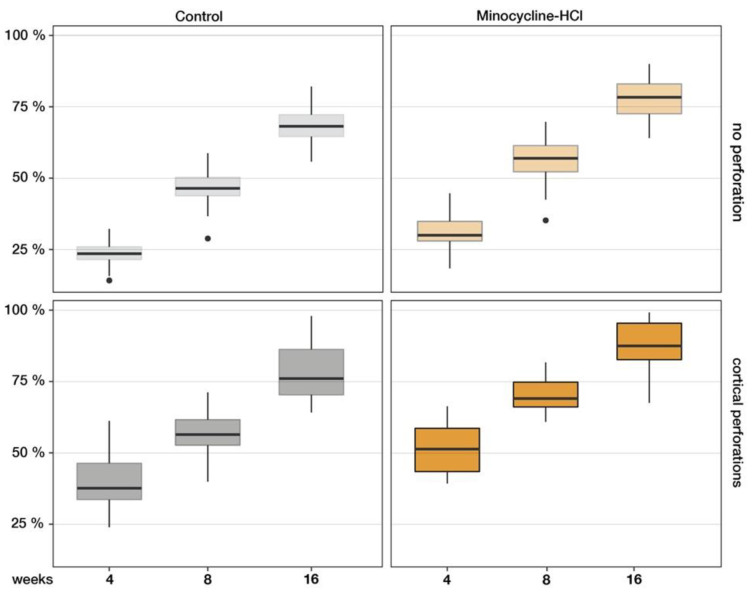
The ratio of mineralised tissues increased continuously in all control and test groups. After 4 months, the non-perforated specimens of the control group and test group MIN showed 68.4 ± 6.7% and 77.6 ± 9.5% mineralisation, respectively. The perforated caps of group P (77.9 ± 6.3%) and group MIN + P (87.2 ± 8.9%) produced the most new lamellar bone, indicating that the combination of marrow opening and systemic minocycline addition had an accelerating and enhancing effect on vertical bone augmentation in the rodent model.

Similar to the 4-week observations, the woven bone proportions were around 25% higher in the perforated groups (I and III) than in the non-perforated groups (C and II). Remodelling of the woven bone scaffold into lamellar bone was ubiquitous (Figure 5); 46.53 ± 8.86% (control group) to 70.08 ± 5.92% (test group III) of the newly generated tissues were mineralised or in the process of mineralisation (Figure 6). As detailed in Table 1, in the minocycline-treated groups, the number of osteocytes was significantly lower (MIN: 507.37 ± 29.64%; MIN + P: 491.88 ± 36.14%) than in the control (C: 615.41 ± 35.61%) and perforated groups (P: 596.38 ± 38.79%).

A difference was still observed between the basal lamellar bone at the bottom of the section and the secondary bone formation centres in the upper regions where the bone growth was the most advanced. Some samples presented a thin layer of periosteum-like tissue interposed between both ossification zones; the surrounding bony surfaces were covered by facing osteoblastic fronts indicating their future fusion with the disappearance of this interposed connective tissue layer.

The de novo lamellar bone appositions outside of the titanium chambers, as described for the 4-week specimens, were still visible, but no additional mineralisation centres, such as those inside the regeneration chambers, were observed.

### 3.3. Sixteen-Week Specimens

From 8 to 16 weeks, the bone formation increase was statistically significant in all test sites. The control sites showed new tissue formation augmented by 137.84 ± 25.79%, which was significantly higher than at 8 weeks. Compared to controls, the gain of newly generated tissue was still significantly increased in test groups I (cortical perforations; 172.61 ± 41.65%; *p* = 0.01) and II (minocycline treated; 182.69 ± 47.27%; *p* < 0.05) and statistically highly different in test group III (minocycline-treated with cortical perforations; 253.94 ± 38.88%; *p* = 0.0001).

Comparing between test groups found that combining cortical perforations with minocycline addition was significantly (*p* < 0.05) more effective than each single technique applied separately.

Non-mineralised tissue areas had regressed in all conditions. Parallel evolution was found for the woven bone fractions in minocycline-untreated samples (woven bone <10%; Table 2). Superior woven bone fractions were observed in the minocycline-treated groups, with 10.13 ± 2.29 % and 11.52 ± 1.97% woven bone in test groups II and III, respectively. Most of the newly generated bone in the control and test conditions was lamellar. The lamellar bone trabeculae contained only a small core of densely stained woven bone surrounded by thick layers of mineralised bone. When the amount of lamellar bone formation was compared between groups, no statistically significant differences were found between the control group (52.62 ± 5.60%) and test group II (minocycline-treated; 59.22 ± 5.47%). Lamellar bone was significantly higher in the perforated groups (test group III: 66.08 ± 8.80%; test group I: 58.87 ± 7.20%). The overall mineralised fractions included around 8–10% of marrow spaces according to the measurements on the original skull bone (Table 2).

**Figure 7:**
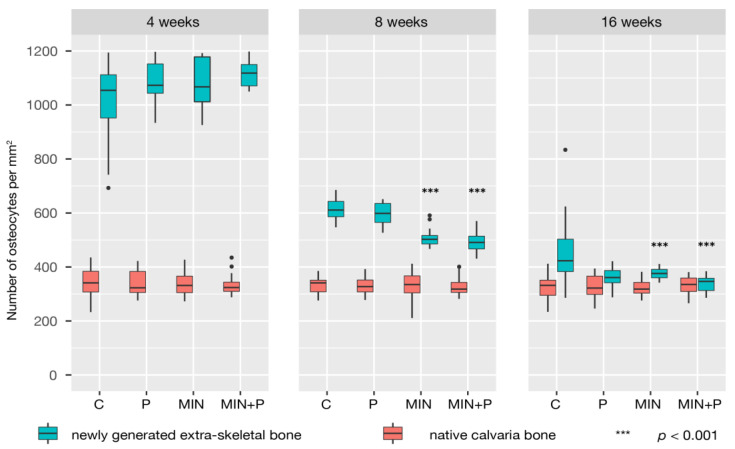
Density of osteocytes in native and newly generated bone. Woven bone contains many more osteocytes than lamellar bone, and the osteocyte density in the newly generated bone (cell number per unit section area of bone—expressed as Oc per mm^2^) fell with its maturation. Notably, minocycline-treated specimens showed fewer osteocytes at 8 weeks than the control and perforated groups.

The osteocyte density (number of Oc per mm^2^) decreased in all groups to values almost 2% above those scored in the mature native skull bone (Figure 7). The bone surfaces were still largely covered by cuboidal osteoblasts, indicating active ossification processes, with the osteoblast size, shape and density hardly changed over time. The highly vascularised connective tissue layer interposed at the base, between the newly formed bone at the surface of the skull and the main core of generated bone inside the chambers, was still present but reduced, and areas of fusion became very frequent. The bone formation outside of the bone generation devices did not significantly vary at the 16-week observation.

## 4. Discussion

Research aiming to optimise bone augmentation techniques is essential. The results of this study demonstrate that predictably augmenting mineralised bone is possible in spaces beyond the skeletal envelope by using a titanium cap. We used this kind of device to provide a defined space for blood clot and new tissue formation. The calvaria provides a site with a considerable area of cortical and medullar bone, easy surgical access and low muscular contraction. The reduced mechanical loads minimise the risk of fracture and make the skull bone an ideal candidate for GBA research [40,41,42].

The conditions must be ideal to allow bone formation beyond the skeletal boundaries. Critical factors for a successful outcome include occlusiveness against cellular invasion, barrier stability, peripheral sealing between barrier and bone, bone blood supply and access to bone-forming cells [3,11,43]. Experimental evidence suggests that fibrous connective tissue proliferation may be a limiting factor for osteogenesis [44]. The titanium caps shield the site of bone generation from this influence and thus allow for bone growth to occur. In the present study, newly generated tissue and mineralised bone were noted in all specimens, regardless of the treatment and even within a month.

Mineralised bone tended to climb along the inner wall of the cap directly on the titanium surface, which is in line with the literature on calvaria [3,45,46], supporting the biocompatibility of titanium and facilitation of bone formation by a solid base. However, the newly generated tissue in our model consisted of evenly distributed mineralised intramembranous bone trabeculae, large fat marrow spaces with numerous blood vessels and areas of woven bone after 16 weeks.

Multiple investigations have reported that perforations of the cortical bone to allow bleeding from the marrow spaces enhances GBA, but research on this topic remains controversial [3,47].

The diameter at which defects are considered to be of critical-size is reported differently in the literature [40]; generally, the perforation created should have a diameter sufficient to allow adequate bleeding and opening of the marrow space with spontaneous repair during the lifetime of the animal. We regularly observed spontaneous and complete repair in both perforated groups, indicating that the diameter of 0.8 mm used in this current study did not create defects to be considered critical-sized [42]. The number and chosen diameter of the holes allowed for bone regrowth to occur and for good histological observations while also preserving the structural integrity of the bone. In the groups C and MIN, the cortical bone was left intact, without surgical opening of the marrow spaces, and no attempt was made to actively fill the completely occlusive bone generation chambers. This procedure yielded a 2.4- to 2.8-times thicker calvaria after 16 weeks, confirming that maintaining a secluded space over the calvaria may be a sufficient condition for substantial bone formation and that marrow opening is not mandatory for new bone formation in a GBA model [11]. The interval of assessment time may also affect the perforation efficacy because the early and late bone healing process may be affected in different ways [48]. Intra-marrow penetrations increased new bone formation at 4 weeks but not at 8 weeks. At 16 weeks, our results show a continuing and improved process of bone formation. This might be explained by the relatively high rate of woven bone observed in the perforated groups up to 8 weeks after surgery. While the approach of cortical perforation to enhance GBA is described differently, there is consensus that intra-marrow penetration is useful to accelerate bone healing [49,50]. This is consistent with our findings that new bone formation is happening at a higher rate in the perforated study groups than in the control group at the fourth month.

A crucial factor in the process of wound healing in general as well as in bone tissue is blood supply, which transports the nutrients and the components, such as osteoprogenitor cells, growth factors and cytokines that mediate the formation of new bone. Angiogenesis occurs during the first phase of ossification. This is a multistep process, with its origins in existing vessels in the bone. Factors released after wounding are responsible for the initiation of blood vessel formation. Therefore, the cortical bone perforations made in the experimental groups I and III improved bleeding and clot formation in the wound area. The subsequent migration of angiogenic and osteogenic progenitor cells into the space may have provided the therapeutic advantage since the proliferation of new capillaries together with the accompanying loose connective tissue represent the source of osteoprogenitor cells and condition for any new bone formation. The detailed histological observations in this study show that new capillaries, together with their perivascular loose connective tissue, were particularly abundant close to the perforations, confirming the quantitative results of the perforated groups. To the best of our knowledge, the number of capillaries and, thus, the capillary network of the newly generated tissues has never been evaluated in a GBA model. Without claim to have focused our research on a specific identification of blood vessels, our results seem in line with other estimates about the capillary density for this type of tissue [51,52].

Our observations of osteocytes fit well with the emerging concept that their number may influence the remodelling activity, formation rate and tissue volume [53,54]. Notably, the number of osteocytes described for the samples at 4 weeks continuously fell over time, reaching values close to those identified in native cranial bone. This suggests that their number decreases as the bone matures, and the rate of matrix synthesis weakens. Great differences in osteocyte number between woven and lamellar bone tissues have been reported in the literature. The osteocyte population in woven bone has been estimated at four to eight times higher than in lamellar bone [55]. A relationship has been described between the cell size of osteoblasts and the rate of matrix synthesis [56]. The size and shape of the osteoblasts in our study were fairly unchanged at the three observation times. An increase in cell size could have indicated a rapid rate of matrix synthesis in woven bone.

Minocycline is a structural isomer of tetracycline and has largely been investigated in bone repair and bone engineering research, mainly due to its ability to inhibit osteoclast genesis and collagenolytic enzymes (responsible for connective tissue degradation and bone resorption) and induce apoptosis of osteoclasts [57]. The inhibition of osteoclastogenesis is undoubtedly a very advantageous feature of this drug since bone manipulation alone during surgical procedures can trigger osteoclast activity [49,50,57].

The minocycline-treated groups showed better tissue fill in short- and long-term specimens when compared to control. The specimens in the minocycline-untreated groups (137 ± 25.79% and 172.61 ± 41.65% at 16 weeks, respectively) had a lower percentage of tissue formation than the minocycline-treated groups (182.69 ± 47.27% and 253.94 ± 38.88% at 16 weeks, respectively).

From 4 to 16 weeks, the non-perforated minocycline group (test group II) presented statistically insignificantly different increasing rates compared to the perforated group (test group I), with around 10% less generated lamellar bone. The combined experimental conditions of group III (minocycline-treated and cortical perforations) showed 2.5-times thicker cranial bone after 8 weeks, remaining unchanged at 16 weeks. This increasing rate was higher than for each technique taken separately, indicating that creating surgical openings by perforating the external cortical bone plate in addition to systemic tetracycline may constitute a favourable environment for substantial bone formation. Significantly higher increasing rates at 8 weeks were also detectable in test group II (minocycline-treated) compared to the control group, confirming findings described by other authors who have suggested that this drug could have stimulated osteogenesis [58,59].

Notably, test groups I and II showed around 57% mineralisation (sum of lamellar bone, marrow and woven bone) of the generated tissue, and test group III 70%, while the control was only 46% mineralised. This is because the samples treated with minocycline had formed the most lamellar bone at 8 weeks (test group II: 43.24 ± 7.06%; test group III: 50.85 ± 5.76%) and the perforated groups had more bone marrow portions (test group I: 5.22 ± 1.42%; test group III: 7.18 ± 3.05%). Our results at 8 weeks also showed that fewer osteocytes were counted in the minocycline-treated groups. Considering that woven bone is a relatively cell-rich tissue, the regression of osteocytes might be due to either differentiation of osteoblasts into lining cells or their death by apoptosis; only some former osteoblasts transition into mature osteocytes completely embedded in the mineralised bone matrix and undergo remodelling processes [60,61]. Bone remodelling includes removal of not only mineralised bone but also residual cells, disappearing thus not from the bone but with the bone.

Our observations were static, and the scope of this work was not to illustrate a dynamic procedure requiring a living model with longitudinal observations. Nevertheless, we can presume that minocycline might have influenced the ossification process by accelerating the maturation of the newly generated osteoid tissues. A possible effect on bone maturation might be of some interest in oral and orthopaedic surgery, but further investigations are required to clarify this.

Minocycline addition resulted in increased density of the newly generated tissue (ratio: mineralised/non-mineralised), with 3.52 and 6.82 times more osseous tissue than non-osseous tissue for test groups II and III, respectively, compared to the minocycline-untreated groups (control: 2.16 times; test group I: 3.45 times). In particular, test group III was the most advanced at 8 weeks, presenting a 3.5-times thicker calvaria plate with a 70% mineralised tissue infill. This group was not only the most active of all the test and control groups, but also kept a great potential for further mineralisation processes, considering the high percentage (11.52 ± 1.97%) of woven bone at 16 weeks. This topic needs further investigation.

## 5. Conclusions

Cortical perforations, by intentionally opening the vessel-rich spongious layer, considerably enhance access for new vessel formation in the regeneration chamber and therefore, favourably affect the outcome of GBA procedures. Minocycline addition results in more complete soft tissue formation in the short-term observations, allowing earlier capillary proliferation and osteoprogenitor cell immigration into the wound area. This remarkably enhances mineralisation, which occurs earlier and results in increased bone density.

These findings highlight the pro-anabolic and anti-catabolic activity of minocycline and may help to explain the statistically highly significant differences in new bone formation in the minocycline series compared to the control series.

It appears clear that cortical perforations involving bone and vessel wounding induce a biologic cascade with activation of some inductive or regulatory factors and new blood vessel formation that leads to new bone formation.

## Figures and Tables

**Table 1:** Capillary, Osteoblast and Osteocyte Histomorphometric Data for Minocycline-HCI and No Minocycline-HCI treated Perforated and Non Perforated Rats.

		No Minocycline-HCI		Minocycline-HCI		Perforation *(p)*	Minocycline *(p)*	Interaction *(p)*
		No Perforation	Bone Perforation	No Perforation	Bone Perforation			
Capillary density (cap/mm^2^)	4	130.0 ± 60.0	138.6 ± 52.7	124.3 ± 55.5	140.1 ± 58.2	0.726	0.740	0.003
	8	104.0 ± 52.4	88.6 ± 38.3	108.3 ± 49.8	139.1 ± 55.4			
	16	88.1 ± 33.7	90.0 ± 43.9	96.3 ± 51.9	136.2 ± 42.6			
Capillary density (cap/mm^2^)	4	48.5 ± 5.8	46.8 ± 8 7	46.7 ± 7.7	48.7 ± 8.1	0.000	0.113	0.065
	8	48.9 ± 6.3	42.6 ± 4.9	47.4 ± 4.5	48.0 ± 4.7			
	16	40.7 ± 5.5	38 9 ± 4.9	47.5 ± 4.7	43 0 ± 4 6			
Capillary density (cap/mm^2^)	4	1106.9 ± 193.1	1166.8 ± 121.6	1203.7 ± 126.5	1154.1 ± 75.5	0.288	0.052	0.383
	8	615.4 ± 35.6	596.4 ± 38.8	507.4 ± 29.6	491.9 ± 36.1			
	16	447.6 ± 103.9	363.5 ± 31.0	376.2 ± 19.5	338.7 ± 29.2			

Notes: Values are means ± *SD.* The *p* values refer to significant main effects identified by a multivariable linear regression.

**Table 2:** Influence of Minocycline-HCI and of cortical perforations on de novo extra-skeletal bone formation.

		No Minocycline-HCI		Minocycline-HCI	
		No Perforation (C)	Perforation (P)	No Perforation (MIN)	Perforation (MIN + P)
4 weeks	**Newly generated tissue rate**	**65.6 ± 15.5**	**73.5 ± 15.0**	99.3 ± 7.6 **	111.6 ± 9.6 *
	**Osseous tissues (Σ)**	**23.6 ± 4.0**	**40.2 ± 9.9**	31.3 ± 6.4 **	51.5 ± 9.1 *
	Woven bone	10.2 ± 2.6	17.3 ± 3.9	12.8 ± 4.2	16.3 ± 5.2
	Lamellar bone	13.0 ± 2.8	22.2 ± 7.5	17.4 ± 6.0	31.8 ± 9.6
	Bone Marrow	0.4 ± 0.7	0.7 ± 11.2	1.2 ± 1.7	3.4 ± 3.3
	**Other**				
	Connective tissue	66.3 ± 5.7	55.2 ± 9.9	63.1 ± 8.7	44.2 ± 9.4
	Granulation tissue/Blood clot	10.1 ± 6.3	4.6 ± 4.2	5.6 ± 4.5	4.3 ± 3.9
8 weeks	**Newly generated tissue rate**	**82.9 ± 21.9**	**120.7 ± 52.1**	116.9 ± 17.4 *	253.6 ± 21.7 **
	**Osseous tissues (Σ)**	**46.5 ± 5.9**	**56.9 ± 7.3**	56.4 ± 8.3 **	70.1 ± 5.9 **
	Woven bone	8.2 ± 1.9	12.7 ± 2.5	9.9 ± 2.7	12.1 ± 2.5
	Lamellar bone	33.8 ± 5.4	39.0 ± 6.1	43.2 ± 7.1	50.9 ± 5.8
	Bone Marrow	4.5 ± 3.4	5.2 ± 1.4	3.3 ± 2.7	7.2 ± 3.1
	**Other**				
	Connective tissue	48.7 ± 6.6	41.1 ± 7.6	40.9 ± 8.6	28.7 ± 6.4
	Granulation tissue/Blood clot	4.7 ± 3.1	2.0 ± 2.6	2.8 ± 2.8	1.3 ± 2.1
16 weeks	**Newly generated tissue rate**	**137.8 ± 25.8**	**172.6 ± 41.7**	**182.7 ± 47.3**	253.9 ± 38.9 *
	**Osseous tissues (Σ)**	**68.4 ± 6.7**	**77.6 ± 9.5**	77.9 ± 6.3 **	87.2 ± 8.9 **
	Woven bone	7.1 ± 1.9	9.7 ± 2.2	10.1 ± 2.3	11.5 ± 2.0
	Lamellar bone	52.6 ± 5.6	58.9 ± 7.2	59.2 ± 5.5	66.1 ± 8.8
	Bone Marrow	8.6 ± 2.2	9.0 ± 2.5	8.5 ± 2.8	9.6 ± 2.7
	**Other**				
	Connective tissue	29.7 ± 7.5	22.0 ± 9.5	21.3 ± 12.8	12.4 ± 9.3
	Granulation tissue/Blood clot	1.9 ± 2.4	0.4 ± 1.0	0.8 ± 1.7	0.4 ± 1.2

Notes: Intervention Min vs. no-Min, values are means ± *SD* (%), * *p* < 0.05, ** *p* < 0.001.

## Data Availability

The data underlying this article will be shared on reasonable request to the corresponding author.
